# Anti-High-Power Microwave RFID Tag Based on Highly Thermal Conductive Graphene Films

**DOI:** 10.3390/ma16093370

**Published:** 2023-04-25

**Authors:** Xueyu Liu, Rongguo Song, Huaqiang Fu, Wei Zhu, Kaolin Luo, Yang Xiao, Bohan Zhang, Shengxiang Wang, Daping He

**Affiliations:** 1 School of Science, Wuhan University of Technology, Wuhan 430070, China; m15927668546@163.com (X.L.); rongguo_song@whut.edu.cn (R.S.); kolin_whut@foxmail.com (K.L.); 261206@whut.edu.cn (Y.X.); 2 Hubei Engineering Research Center of RF-Microwave Technology and Application, Wuhan University of Technology, Wuhan 430070, China; 3 School of Materials Science and Engineering, Wuhan University of Technology, Wuhan 430070, China; fuhuaqiang@whut.edu.cn; 4 School of Mathematical and Physical Sciences, Wuhan Textile University, Wuhan 430073, China; zhuwei@wtu.edu.cn (W.Z.); shxwang@wtu.edu.cn (S.W.); 5 State Key Laboratory of New Textile Materials and Advanced Processing Technologies, Wuhan Textile University, Wuhan 430200, China

**Keywords:** RFID tag, anti-high-power microwave, graphene films, highly thermal conductivity, UHF

## Abstract

In this paper, a radio frequency identification (RFID) tag is designed and fabricated based on highly electrical and thermal conductive graphene films. The tag operates in the ultrahigh-frequency (UHF) band, which is suitable for high-power microwave environments of at least 800 W. We designed the protection structure to avoid charge accumulation at the antenna’s critical positions. In the initial state, the read range of the anti-high-power microwave graphene film tag (AMGFT) is 10.43 m at 915 MHz. During the microwave heating experiment, the aluminum tag causes a visible electric spark phenomenon, which ablates the aluminum tag and its attachment, resulting in tag failure and serious safety issues. In contrast, the AMGFT is intact, with its entire read range curve growing and returning to its initial position as its temperature steadily decreases back to room temperature. In addition, the proposed dual-frequency tag further confirms the anti-high-power microwave performance of graphene film tags and provides a multi-scenario interactive application.

## 1. Introduction

RFID technology belongs to the sensing layer of the Internet of Things (IoT), which is a non-contact, real-time, quick, secure, and accurate automatic identification technology that is used extensively in the monitoring of logistics, unmanned retail, healthcare, and other fields [1,2,3,4,5]. However, as high-power microwave research continues, military and medical fields are flooded with electromagnetic waves, and accordingly, RFID tags in this application need to be able to work safely under long-term exposure to microwave radiation. Current metal antenna-based RFID tags cannot be worked in high-power microwave environments such as microwave ovens. This is because the strong electromagnetic field causes charge accumulation on the antenna’s surface and high-speed friction between molecules generates high heat. It exceeds the material’s limit (aluminum foil thermal conductivity is 214 W·m^−1^·K^−1^, ignition point is 500 °C), causing arc discharges, sparks, and even deflagration phenomena, which can damage the tags and other items and pose serious safety risks [6,7,8]. Additionally, the tip effect is a significant contributor to arc discharge. The more curved the arc and the denser the charge on the metal surface, the more obvious the tip effect is. Most commercial tag antennas are dipole-like bending line forms, so the arc discharge is easy to occur at the parts of the antenna and chip binding and the antenna bend. The current design approaches for RFID tags that apply to the high-power electromagnetic field are as follows: i. Covering the tag antenna with high dielectric coefficient materials and increasing capacitance to allow the antenna conductor to hold more charges; ii. Setting up a sacrificial or shielded conductor structure to disperse charge accumulation; and iii. Setting up a microwave reflection or heat sink connection structure at the chip to reflect or absorb heat [9]. These techniques are based on conventional metal antennas, which call for the addition of extra structures to disperse or inhibit charge buildup, resulting in complex fabrication procedures and high processing costs, thus making them unsuitable for mass production and large-scale applications. The market is in urgent need of a new material that is both electrically and thermally conductive to achieve the replacement of traditional metal antennas in the fabrication of microwaveable RFID tags.

With its special two-dimensional structure and phonon scattering mechanism, monolayer graphene has a thermal conductivity as high as 5300 W·m^−1^·K^−1^. However, monolayer graphene does not achieve the expected performance in practical applications owing to its thickness of only 0.34 nm and its non-self-supporting structure, which makes it difficult to transfer. Most importantly, the heat flux carrying capacity is limited and cannot achieve the expected performance in practical applications [10,11,12,13]. Therefore, in this paper, to obtain graphene films with outstanding flexibility, conductivity (10^6^ S·m^−1^), and thermal conductivity (1300 W·m^−1^·K^−1^), graphene oxide (GO) films were prepared by vacuum filtering and evaporative drying, then annealed at high temperatures and further statically calendered [14,15,16]. The specific preparation method can be found in the published articles of our research group [17,18,19,20]. It is worth mentioning that the graphene films can be prepared on large scales in rolls which effectively reduces the raw material cost. In this work, we designed and fabricated the AMGFT using highly thermal conductive graphene films. Firstly, we introduced the preparation and characterization of graphene films and explain the mechanism underlying their high thermal conductivity. Then combine the current distribution on the surface of the graphene film antenna with the design and optimization of the antenna structure. The graphene film tag was manufactured and its performance was tested, the results demonstrate graphene film has more advantages compared to metallic materials in the use of a high-power microwave. In addition, we proposed the dual-frequency tag providing a multi-scenario interactive application.

## 2. Preparation of Graphene Film

We purchased GO from Wuhan Hanxi Technology Co. LTD (Wuhan, China) and used a five-step process to fabricate the graphene films. Firstly, GO and deionized water are put into a homogenizing mixer to obtain GO aerogel (50 mg/mL). Secondly, GO aerogels are scraped into GO films (2 mm) on polyethylene terephthalate substrates. Thirdly, the GO films are dried at 200 °C in an oven for 6 h. Then the obtained dried GO films were annealed at 2850 °C for 2 h under an argon atmosphere. Finally, under a compressed pressure of 300 MPa, graphene films can be obtained.

**Figure 1:**
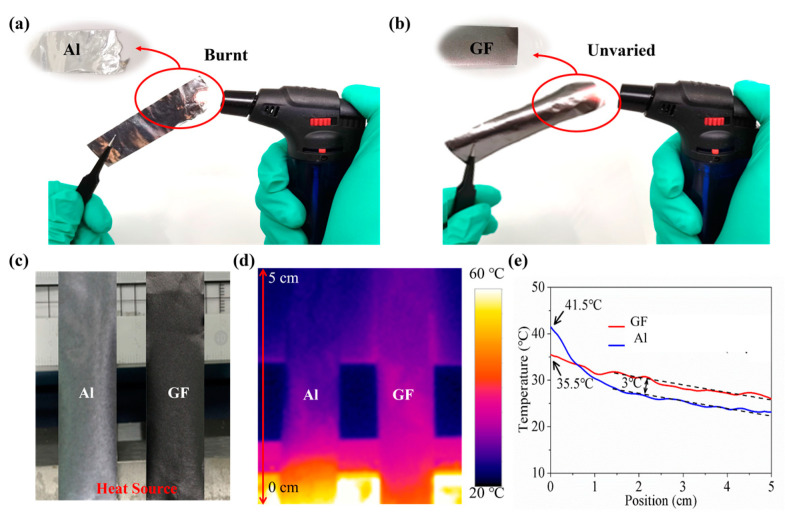
High-temperature experiment of (**a**) aluminum and (**b**) graphene. (**c**) The platform for thermal conductivity test. (**d**) The image of thermal infrared camera. (**e**) The surface temperature of strip-shaped aluminum foil and graphene film after heating at 60 °C for 1 min.

Next, we investigated the flammability and thermal conductivity of graphene films and aluminum foil as shown in Figure 1a–e. Two identical strips of graphene film and aluminum foil were heated by a heat source of 60 °C at one end simultaneously. After 2 min, the surface temperatures of the sample strips were measured by the thermal infrared camera Testo 865. The surface temperature at the source end of the graphene film strip was 35.5 °C, which was 6 °C lower than that of aluminum. Furthermore, the GF strip was found to have a smaller temperature gradient than an aluminum strip. From the position of 1.5 cm, the surface temperature of the graphene film strip was almost 3 °C higher than the aluminum [21]. These results indicate that the graphene films have superior thermal spreading performance, which show great advantages in high-temperature resistant applications for RFID.

## 3. Antenna Design

**Figure 2:**
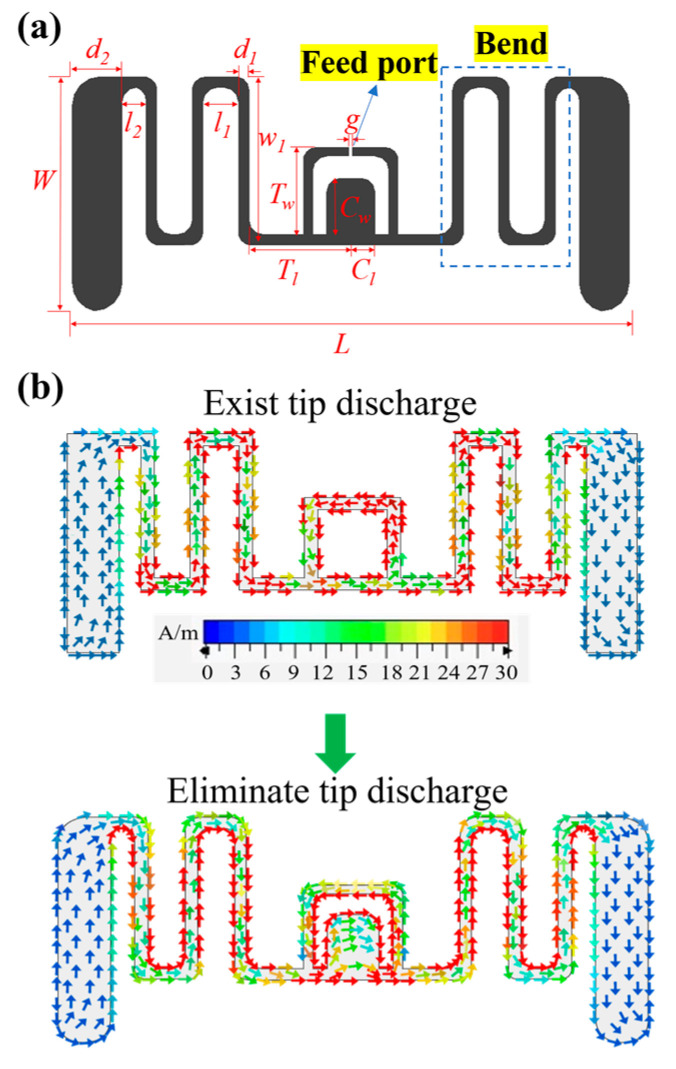
(**a**) The geometry and parameter diagram of the proposed antenna. (**b**) The simulated current distribution of antenna surface at 915 MHz with (up) and without (down) high-temperature resistant design.

We further optimize the antenna structure to avoid charge buildup at critical antenna locations to prevent the antenna from arcing and detonation during heating. The geometry of the proposed AMGFT is shown in Figure 2a. We constructed two models using CST simulation software, solved Maxwell’s equations by finite integration method, meshed the parameter domain with a rule of 15 cells per wavelength, and obtained the simulation current diagram of the antenna. The simulated current distribution of the antenna surface with and without a high-temperature resistant design is shown in Figure 2b. The arrows indicate the flow path of the surface current, and the intensity of the magnetic field caused by the surface current is distinguished by color. It can be seen from the current distribution that the current density is high at the antenna port and the antenna bend without the heat-resistant design. As a result, it may burn the chip’s internal integrated circuit (IC) at the antenna port and also cause tip discharge at the antenna bend. To avoid these, we loaded a central rectangular block at the bottom of the antenna port and smoothed all the bends and sharp corners at the end of the antenna. The center rectangular block is loaded to increase the current flow path and effectively reduce the current density and magnetic field strength at the antenna port, which can reduce the heat circulation of the chip. The tip corner smoothing process also reduces the magnetic field strength, thus achieving the effect of avoiding tip discharge.

In order to conjugately match the complex impedance of the antenna and the chip at 915 MHz, parameter optimization has been carried out (Figure S1 and Figure S2). It can be concluded that the end loading area primarily influences the real part of the antenna input impedance, and the size of the T-matching structure has a greater impact on the imaginary part of the antenna impedance. The optimized antenna parameters are shown in Table 1.

## 4. AMGFT Tests and Results

Instead of conventional metal etching technology, we employ the LPKF laser machine to engrave the antenna outline, which has the advantages of high precision, simple process, and environmental protection [22,23]. The carved antenna is transferred to the high-temperature resistant substrate polyimide (PI) [24] and the chip is inverted and encapsulated in the antenna port using a semi-automatic binding machine (Figure S3a). It should be noted that besides loading the center rectangular block to reduce the heat circulation of the chip during the antenna structure design, we cover the chip with Hasuncast 735 vinyl which is purchased at Hazen brand glue shop to shield it from the strong electromagnetic field environment (Figure S3b), which is crucial for high-temperature protection.

The read range of the tag is an important performance indicator, which can be derived from the Friis equation as follows:

(1) $$R_{tag}=\frac{\lambda }{4\pi }\sqrt{\frac{P_{r}G_{r}G_{t}}{P_{t}}}=\frac{\lambda }{4\pi }\sqrt{\frac{P_{r}G_{r}G_{t}\tau }{P_{c}}}$$

where *τ* is the power transmission coefficient, *P_r_* and *G_r_* are the transmitted power and realized gain of the reader, *P_t_*, *G_t_* are the incident power and realized gain of the tag. *EIRP* = *P_r_G_r_* is the equivalent isotropic radiated power of the reader, which is the value specified for each region. *P_c_* = *τP_t_* is the least power needed to wake up the device, that is, the chip sensitivity is also a fixed value, and the R6 chip sensitivity chosen for this paper is −20 dBm. Therefore, within a certain range, the read range of RFID tags can be enhanced by increasing *τ* (the ideal value is 1) and *G_t_*.

**Figure 3:**
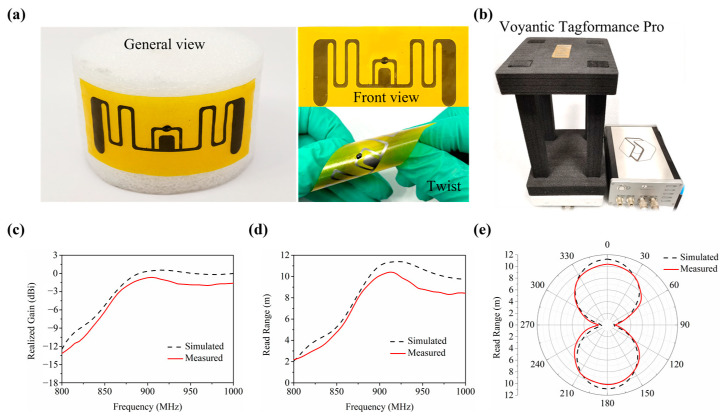
(**a**) Photographs showing the AMGFT including the front view and twisting test. (**b**) Experimental setup for read range test. (**c**,**d**) Simulated and measured antenna (**c**) realized gain, (**d**) tag read range, and (**e**) radiation patterns in terms of AMGFT read range at 915 MHz in E-plane.

Figure 3a shows the digital photograph of the proposed AMGFT in curled status, indicating excellent flexibility. The simulated and measured curves of the realized gain and the read range (measured using the setup in Figure 3b) are shown in Figure 3c,d, the measured value of antenna realized gain exceeds −5.01 dBi in the whole UHF band (860 MHz to 960 MHz), slightly lower than the simulated value. The measured read range of the tag is greater than 5.79 m in the whole UHF band, with a maximum value of 10.43 m at 915 MHz, which meets the practical application requirements of the tag. Figure 3e shows the simulated and measured radiation patterns in terms of AMGFT read range at 915 MHz in E-plane, which is a typical dipole-like radiation pattern.

**Figure 4:**
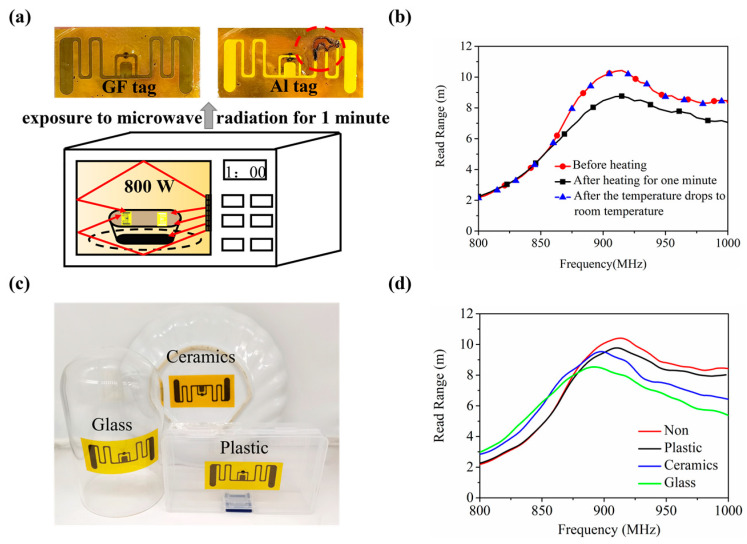
(**a**) Heating the AMGFT and aluminum tag in the microwave oven. (**b**) Variation of AMGFT read range before and after heating. (**c**) AMGFTs attached to three daily microwaveable materials. (**d**) Read range of AMGFT with and without substrate after microwave heating.

Take the heating experiment in a microwave oven as an example we investigate the applicability of AMGFT in high-power microwave environments. We attached the AMGFT and the aluminum tag to the plastic box, being exposed to the high-power microwave environment with a frequency of 2.45 GHz and a power of 800 W. During the heating process, the aluminum tag will spark within 5 s. The experimental process is shown in Figure 4a and Video S1. The arc discharge occurs at the antenna bend, and the aluminum antenna is ablated to break resulting in tag failure. In contrast, the AMGFT is intact and can work stably in a microwave oven. As seen from Figure 4b, after heating, the read range of AMGFT decreases slightly but returns to the initial state as the temperature gradually cools down. This is due to the significant change in the chip input impedance with the increase in temperature. This change is, however, reversible in a certain range: once the tag temperature drops back to room temperature, its read range is restored, ensuring the microwave stability of the designed tag.

Next, to verify the general applicability of AMGFT, we selected three common microwave-safe materials (plastic, ceramic, and glass) as substrates of AMGFT, as shown in Figure 4c. Figure 4d depicts the read range curves of AMGFT with and without substrate after microwave heating. After heating for 1 min at 800 W in the same environment, the substrate effect on the tag read range follows the order of glass > ceramic > plastic and both the ceramic and glass substrates shift the maximum read range frequency to a lower value. These phenomena can be explained by the effect of changing the dielectric constant and the bending angle of the substrate on the read range. When the bending angle of the substrate is the same, the higher the dielectric constant of the substrate material, the greater the influence on the complex impedance of the antenna and the read range of the tag; with the same dielectric constant of substrate material, the tag read range decreases with the increase in bending angle. The dielectric constant of glass is lower than that of ceramic, but it requires bending deformation of the tag to be attached to its sidewall. The combination of these two factors makes the read range of AMGFT on the bottle substrate lower than that on the ceramic substrate.

**Figure 5:**
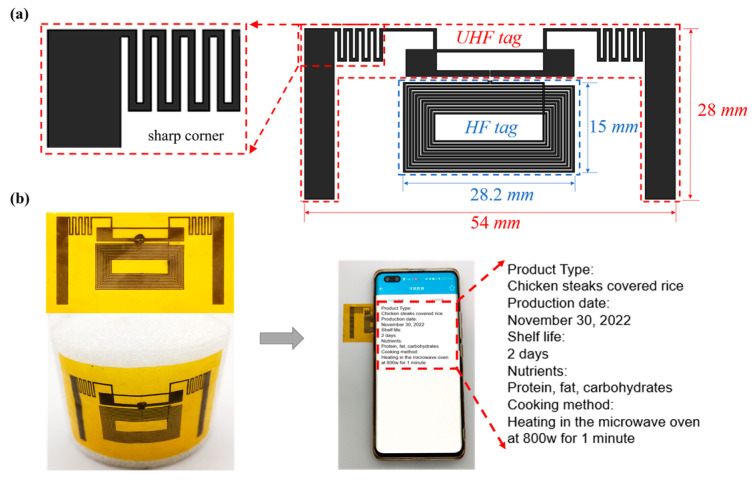
(**a**) The geometry and details of the dual-band AMGFT. (**b**) The demonstration of the tag as an electronic smart label compatible with NFC devices.

In fact, most people do not have special RFID readers to read the information on RFID tags. Nowadays, with the increasing prevalence of NFC-equipped mobile phones, people can use NFC to obtain chip information anytime, anywhere. Therefore, the dual-band anti-high-power tag is demanded. As shown in Figure 5a, the proposed dual-band tag contains two modules, UHF tag measuring 28 mm × 54 mm and a high frequency (HF) tag measuring 15 mm × 28.2 mm, which are linked with a dual-band chip. By bringing the NFC-equipped mobile phone near the tag on the lunch box, we can promptly obtain the information stored in the tag such as the type of food as shown in Figure 5b. As most RFID tag models in the market contain sharp-angle structures. So the sharp-angle part of the antenna is reserved in the design of the dual-frequency tag in order to reduce the design cost and processing difficulty. After heating in the microwave oven, the dual-band tag, which excludes the advantages of structural improvements, still worked well, further validating the advantages of graphene in the thermal field.

## 5. Conclusions

In conclusion, the anti-high-power microwave UHF RFID tag based on highly thermal conductive graphene films is designed and fabricated in this paper. The microwave stability of AMGFT was verified by the contrast experiment of exposing the AMGFT and aluminum tag in the high-power microwave environment with a frequency of 2.45 GHz and a power of 800 W for 1 min. Then, the read range test was carried out on attachments with different dielectric constants and bending angles, which provides the possibility for more application scenarios. What is more, the proposed dual-band tags preserving sharp corners make the design and use of tags more intelligent and convenient and further confirmed the advantage of graphene in thermodynamics. These results extend the application field of tags and provide a solution for tags working in high-emission fields.

## Figures and Tables

**Table 1:** Optimized parameters of the proposed antenna.

Parameter	Values (mm)	Parameter	Values (mm)
*L*	58.64	*T*_l_	16.06
*W*	25.00	*T*_w_	9.00
*l*_1_	3.70	*C*_l_	3.00
*l*_2_	2.50	*Cw*	5.00
*w*_1_	18.00	*d1*	1.20
*g*	0.15	*d2*	5.00

## Data Availability

Not applicable.

## Supplementary Materials

The following supporting information can be downloaded at: https://www.mdpi.com/article/10.3390/ma16093370/s1, Figure S1: (a,b) Variations of changing the T-matching ring length (*T_l_*) on (a)simulated antennas impedance and (b) return loss response vs frequency. (c,d) Variations of (c) antennas impedance and (d) simulated return loss response of antennas vs frequency for different T-matching ring width (*T_w_*); Figure S2: (a,b) Variations of changing the end loading width (*d_2_*) on (a) simulated antennas impedance and (b) return loss response vs frequency. (c,d) Variations of (c) antennas impedance and (d) simulated return loss response of antennas vs frequency for different end loading length (*W*); Figure S3: (a) The device of flip chip bonding. (b) Hasuncast 735 vinyl; Video S1: Experimental demonstration of the heating of tags in a microwave oven. In this movie, the GFMT and the aluminum tag are attached to the lunch box and then placed in a microwave oven heating for one minute at 800 W. During the heating process, the aluminium tag will spark within 5 seconds, burning the tag and its attachments. While the GFMT is intact and the maximum read range after heating was measured to remain at 10 m.

## References

[1] Ramesh G., RamaMurthy B. (2020). Implementation of High Performance UHF-RFID for Logistics Management System. J. Eng. Res. Appl..

[2] Unterhuber A.R., Iliev S., Biebl E.M. (2020). Estimation Method for High-Speed Vehicle Identification with UHF RFID Systems. IEEE J. Radio Freq. Identif..

[3] Alvarez-Narciandi G., Motroni A., Pino M.R., Buffi A., Nepa P. (2019). A UHF-RFID Gate Control System Based on a Recurrent Neural Network. IEEE Antennas Wirel. Propag. Lett..

[4] Arboleya A., Laviada J., Alvarez-Lopez Y., Las-Heras F. (2021). Real-Time Tracking System Based on RFID to Prevent Worker–Vehicle Accidents. IEEE Antennas Wirel. Propag. Lett..

[5] Barge P., Biglia A., Comba L., Ricauda A.D., Tortia C., Gay P. (2020). Radio Frequency Identification for Meat Supply-Chain Digitalisation. Sensors.

[6] Thakur M., Møen-Tveit G., Vevle G., Yurt T. (2020). A Framework for Traceability of Hides for Improved Supply Chain Coordination. Comput. Electron. Agric..

[7] Taoufik S., Dherbecourt P., El-Oualkadi A., Temcamani F. (2017). Reliability and Failure Analysis of UHF RFID Passive Tags under Thermal Storage. IEEE Trans. Device Mater. Reliab..

[8] Saarinen K., Frisk L. (2013). Reliability Analysis of UHF RFID Tags under a Combination of Environmental Stresses. IEEE Trans. Device Mater. Reliab..

[9] Ria A., Michel A., Singh R.K., Franchina V., Bruschi P., Nepa P. (2020). Performance Analysis of a Compact UHF RFID Ceramic Tag in High-Temperature Environments. IEEE J. Radio Freq. Identif..

[10] Wang N., Samani M.K., Li H., Dong L., Zhang Z., Su P., Chen S., Chen J., Huang S., Yuan G. (2018). Tailoring the Thermal and Mechanical Properties of Graphene Film by Structural Engineering. Small.

[11] Kumar P., Shahzad F., Yu S., Hong S.M., Kim Y.H., Koo C.M. (2015). Large-Area Reduced Graphene Oxide Thin Film with Excellent Thermal Conductivity and Electromagnetic Interference Shielding Effectiveness. Carbon.

[12] Huang P., Li Y., Yang G., Li Z.X., Li Y.Q., Hu N., Fu S.Y., Novoselov K.S. (2021). Graphene Film for Thermal Management: A Review. Nano Mater. Sci..

[13] Malekpour H., Chang K.H., Chen J.C., Lu C.Y., Nika D.L., Novoselov K.S., Balandin A.A. (2014). Thermal Conductivity of Graphene Laminate. Nano Lett..

[14] Fan C., Wu B., Song R., Zhao Y., Zhang Y., He D. (2019). Electromagnetic Shielding and Multi-Beam Radiation with High Conductivity Multilayer Graphene Film. Carbon.

[15] Zhang J., Song R., Zhao X., Fang R., Zhang B., Qian W., Zhang J., Liu C., He D. (2020). Flexible Graphene-Assembled Film-Based Antenna for Wireless Wearable Sensor with Miniaturized Size and High Sensitivity. ACS Omega.

[16] Wang Z., Mao B., Wang Q., Yu J., Dai J., Song R., Pu Z., He D., Wu Z., Mu S. (2018). Ultrahigh Conductive Copper/Large Flake Size Graphene Heterostructure Thin-Film with Remarkable Electromagnetic Interference Shielding Effectiveness. Small.

[17] Song R., Jiang S., Hu Z., Fan C., Li P., Ge Q., Mao B., He D. (2022). Ultra-High Conductive Graphene Assembled Film for Millimeter Wave Electromagnetic Protection. Sci. Bull..

[18] Song R., Wang Z., Zu H., Chen Q., Mao B., Wu Z.P., He D. (2021). Wideband and Low Sidelobe Graphene Antenna Array for 5G Applications. Sci. Bull..

[19] Zhang B., Zhang C., Wang Y., Wang Z., Liu C., He D., Wu Z.P. (2021). Flexible Anti-Metal RFID Tag Antenna Based on High-Conductivity Graphene Assembly Film. Sensors.

[20] Zhang B., Wang Z., Song R., Fu H., Zhao X., Zhang C., He D., Wu Z.P. (2021). Passive UHF RFID Tags Made with Graphene Assembly Film-Based Antennas. Carbon.

[21] Zabek D., Seunarine K., Spacie C., Bowen C. (2017). Graphene Ink Laminate Structures on Poly (Vinylidene Difluoride) (PVDF) for Pyroelectric Thermal Energy Harvesting and Waste Heat Recovery. ACS Appl. Mater. Interfaces.

[22] Song R., Zhao X., Wang Z., Fu H., Han K., Qian W., Wang S., Shen J., Mao B., He D. (2020). Sandwiched Graphene Clad Laminate: A Binder-Free Flexible Printed Circuit Board for 5G Antenna Application. Adv. Eng. Mater..

[23] Song R., Mao B., Wang Z., Hui Y., Zhang N., Fang R., Zhang J., Wu Y., Ge Q., Kostya S.N. (2023). Comparison of Copper and Graphene-Assembled Films in 5G Wireless Communication and THz Electromagnetic-Interference Shielding. Proc. Natl. Acad. Sci. USA.

[24] Gao X., Lin L., Liu Y., Huang X. (2015). LTPS TFT Process on Polyimide Substrate for Flexible AMOLED. J. Disp. Technol..

