# Supplementary material for: Anti-High-Power Microwave RFID Tag Based on Highly Thermal Conductive Graphene Films
Source: Materials (Basel). 2023 Apr 25;16(9):3370. doi: 10.3390/ma16093370 (PMC10180182; doi:10.3390/ma16093370)
Supplement: Supplementary file 1 [file materials-16-03370-s001.zip › Materials-Supplementary Material-Xueyu Liu.pdf]

Supporting Information

Anti-high-power Microwave RFID Tag Based on Highly Thermal Conductive Graphene Films

Xueyu Liu † [1,2], Rongguo Song † [1,2], Huaqiang Fu [1,2], Wei Zhu [3,4] Kaolin Luo [1,2], Yang Xiao [1,2], Bohan Zhang [3,4,*], Shengxiang Wang [3,4] and Daping He [1,2,*]

[1] School of Science, Wuhan University of Technology, Wuhan, China
[2] Hubei Engineering Research Center of RF-Microwave Technology and Application, Wuhan University of Technology, Wuhan, China
[3] School of Mathematical and Physical Sciences, Wuhan Textile University, Wuhan, China
[4] State Key Laboratory of New Textile Materials and Advanced Processing Technologies, Wuhan Textile University, Wuhan, China
* Correspondence: bhzhang@wtu.edu.cn (B.Z.); hedaping@whut.edu.cn (D.H.)

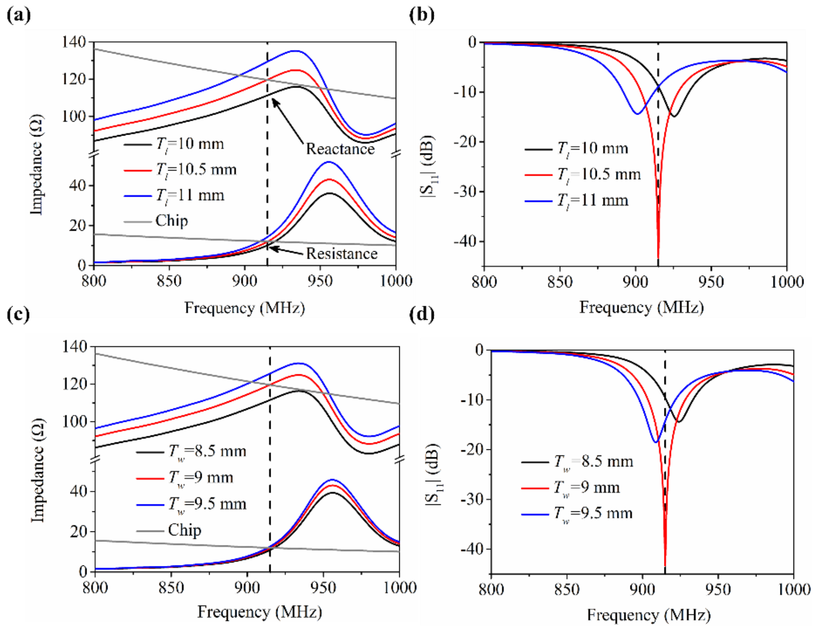

Figure S1 (a-b) Variations of changing the T-matching ring length (T_l) on (a)simulated antennas impedance and (b) return loss response vs frequency. (c-d) Variations of (c) antennas impedance and (d) simulated return loss response of antennas vs frequency for different T-matching ring width (T_w).

The length T_l and width T_w of the T-matching ring were simulated and analyzed in order to study the size effect of T-matching structure on antenna impedance. As shown in Figure S1 (a-d), keeping other parameters constant and scanning T_l and T_w with steps of 0.5 mm, the simulated values of the real and imaginary components of the antenna complex impedance grow as T_l and T_w increase, and the resonant frequencies of the antenna are shifted to the lower frequency. The size of the T-matching structure has a greater impact on the imaginary part of the antenna impedance than the real part. The intersection of the simulated value of the antenna complex impedance (red line) and the chip conjugate complex impedance (thin black line) at 915 MHz indicates that the simulated value of the antenna complex impedance and the chip complex impedance have attained conjugate matching when T_l = 10.5 mm and T_w = 9 mm.

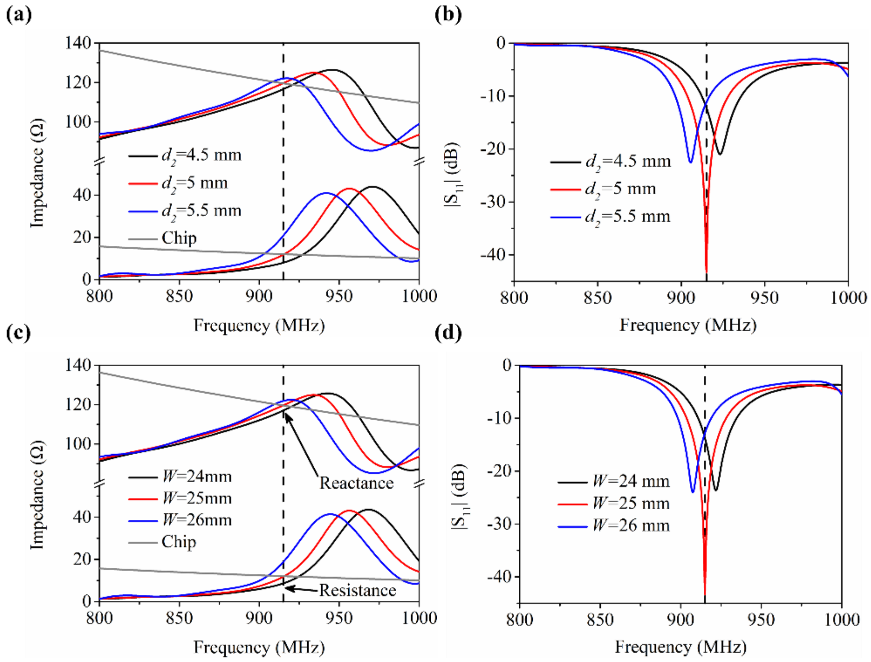

Figure S2 (a-b) Variations of changing the end loading width (*d*2) on (a) simulated antennas impedance and (b) return loss response vs frequency. (c-d) Variations of (c) antennas impedance and (d) simulated return loss response of antennas vs frequency for different end loading length (*W*).

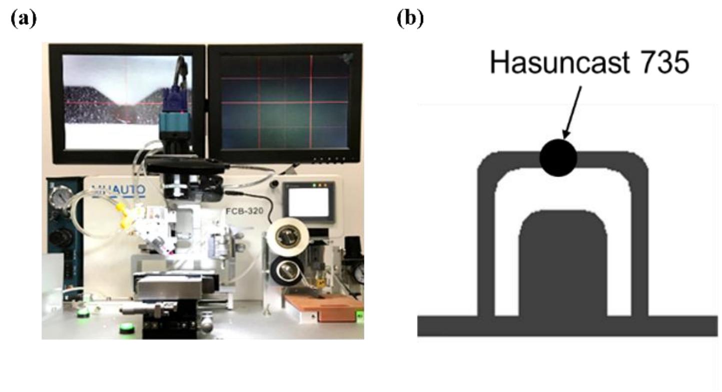

Figure S3 (a) The device of flip chip bonding. (b) Hasuncast 735 vinyl.

Video S1 Experimental demonstration of the heating of tags in a microwave oven. In this movie, the GFMT and the aluminum tag are attached to the lunch box and then placed in a microwave oven heating for one minute at 800 W. During the heating process, the aluminium tag will spark within 5 seconds, burning the tag and its attachments. While the GFMT is intact and the maximum read range after heating was measured to remain at 10 m.
